# Racial and Socioeconomic Equity of Tecovirimat Treatment during the 2022 Mpox Emergency, New York, New York, USA

**DOI:** 10.3201/eid2911.230814

**Published:** 2023-11

**Authors:** Maura K. Lash, Ned H. Latham, Pui Ying Chan, Mary M.K. Foote, Elizabeth A. Garcia, Matthew F. Silverstein, Marcia Wong, Mark Alexander, Karen A. Alroy, Lovedeep Bajaj, Kuan Chen, James Steele Howard, Lucretia E. Jones, Ellen H. Lee, Julian L. Watkins, Tristan D. McPherson

**Affiliations:** New York City Department of Health and Mental Hygiene, Queens, New York, USA (M.K. Lash, N.H. Latham, P.Y. Chan, M.M.K. Foote, E.A. Garcia, M.F. Silverstein, M. Wong, M. Alexander, K.A. Alroy, L. Bajaj, K. Chen, J.S. Howard, L.E. Jones, E.H. Lee, J.L. Watkins, T.D. McPherson);; Columbia University, New York, New York, USA (N.H. Latham)

**Keywords:** mpox, health equity, racism, tecovirimat, emergencies, sexually transmitted infections, viruses, New York City, New York, United States

## Abstract

We assessed tecovirimat treatment equity for 3,740 mpox patients in New York, New York, USA, during the 2022 mpox emergency; 32.4% received tecovirimat. Treatment rates by race/ethnicity were 38.8% (White), 31.3% (Black/African American), 31.0% (Hispanic/Latino), and 30.1% (Asian/Pacific Islander/other). Future public health emergency responses must prioritize institutional and structural racism mitigation.

In May 2022, mpox cases were detected in multiple non–mpox-endemic countries. Increasing numbers of cases worldwide, primarily within sexual networks of gay and bisexual men, prompted the World Health Organization to declare a public health emergency in July 2022 (*1*). Although mpox-associated deaths have been infrequent (*1*), mpox can cause considerable illness (*2*).

No antiviral medications have been approved for mpox treatment in the United States (*3*). However, an Expanded Access Investigational New Drug (EA-IND) protocol held by the Centers for Disease Control and Prevention enabled health providers to prescribe tecovirimat for persons with mpox in the United States. The New York City (NYC) Department of Health and Mental Hygiene (DOHMH) coordinated the distribution of tecovirimat to hospitals and a single partner pharmacy that delivered medication to NYC addresses cost-free for patients. DOHMH provided technical support to prescribers and intentional outreach to federally qualified health centers and safety-net health systems.

In the United States, mpox disproportionately affects Hispanic/Latino and Black persons (*4*,*5*), consistent with well-established inequities in healthcare access and outcomes because of interpersonal, institutional, and structural racism (*6*–*8*). For example, racial inequities in access to HIV preexposure prophylaxis are well-documented and affect similar populations (*9*). We explored racial and socioeconomic inequities in tecovirimat treatment of mpox in NYC.

## The Study

We included all NYC residents who had a positive nonvariola orthopoxvirus (probable case) or mpox (confirmed case) test reported to DOHMH during May 19–October 29, 2022. We collected data on age, gender, race/ethnicity, sexual orientation, and residential addresses during standardized interviews. We obtained tecovirimat treatment data from provider reports via a mandatory REDCap survey and from partner pharmacy dispensing records. We matched cases with treatment data by using names, dates of birth, and postal (ZIP) codes. For persons treated before their first positive test, we used the treatment date instead of diagnosis date. We excluded persons who were treated but never had a reported positive test.

We calculated descriptive statistics for selected demographic characteristics (Table), both overall and according to treatment status. We assessed differences by using χ^2^ tests or *t*-tests. We included unknown values as separate categories under race/ethnicity and sexual orientation. We calculated cumulative changes in percentages of tecovirimat-treated persons in 2-week intervals according to race/ethnicity and neighborhood poverty level (defined as the percentage of residents in the patient’s ZIP code living below the federal poverty level according to the American Community Survey [https://www.census.gov]). We categorized neighborhoods into 4 groups: low poverty, <10%; medium, 10%–19.9%; high, 20%–29.9%; and very high, >30%. We performed analyses by using SAS version 9.4 (SAS Institute, https://www.sas.com) and R version 4.2.3 (The R Project for Statistical Computing, https://www.r-project.org). We considered a p value <0.05 statistically significant. DOHMH’s Institutional Review Board deemed this evaluation to be public health surveillance.

**Table:** Characteristics of persons with mpox according to treatment status in study of racial and socioeconomic equity of tecovirimat treatment during 2022 mpox emergency, New York City, New York, USA, May 19, 2022–October 29, 2022*

Characteristics	Overall	Tecovirimat treatment	No treatment	p value
Total no. persons	3,740 (100)	1,213 (32.4)	2,527 (67.6)	NA
Median age (IQR)	35 (12)	36 (11)	35 (12)	ND
Mean age (SD)	37 (9.4)	37 (9)	36 (9.5)	0.004
Age groups, y				<0.001
0–24	255 (6.8)	55 (4.5)	200 (7.9)	
25–34	1,527 (40.8)	471 (38.8)	1,056 (41.8)	
35–44	1,265 (33.8)	449 (37.0)	816 (32.3)	
45–54	507 (13.6)	177 (14.6)	330 (13.1)	
55–64	166 (4.4)	55 (4.5)	111 (4.4)	
>65	20 (0.5)	6 (0.5)	14 (0.6)	
Gender†				0.01
Men	3,516 (94.2)	1,133 (93.5)	2,383 (94.6)	
Nonbinary/gender queer	52 (1.4)	22 (1.8)	30 (1.2)	
Transgender men or women	70 (1.9)	33 (2.7)	37 (1.5)	
Women	93 (2.5)	24 (2.0)	69 (2.7)	
Unknown	9	1	8	
Sexual orientation				<0.001
LGBQ+	2,409 (64.4)	857 (70.7)	1,552 (61.4)	
Straight	296 (7.9)	61 (5.0)	235 (9.3)	
Unknown‡	1,035 (27.7)	295 (24.3)	740 (29.3)	
Race/ethnicity§				<0.001
Asian/Pacific Islander/other	196 (5.2)	59 (4.9)	137 (5.4)	
Black/African American	1,017 (27.2)	318 (26.2)	699 (27.7)	
Hispanic/Latino	1,294 (34.6)	400 (33.0)	894 (35.4)	
White	847 (22.7)	329 (27.1)	518 (20.5)	
Unknown‡	386 (10.3)	107 (8.8)	279 (11.0)	
Borough of residence				<0.001
Bronx	729 (19.5)	259 (21.4)	470 (18.6)	
Brooklyn	888 (23.7)	299 (24.6)	589 (23.3)	
Manhattan	1,480 (39.5)	504 (41.5)	976 (38.7)	
Queens	596 (15.9)	139 (11.5)	457 (18.1)	
Staten Island	45 (1.2)	12 (1.0)	33 (1.3)	
Unknown	2	0	2	
Neighborhood poverty level¶				0.53
Low	579 (15.5)	177 (14.6)	402 (16.0)	
Medium	1588 (42.6)	508 (41.9)	1080 (42.9)	
High	894 (24.0)	303 (25.0)	591 (23.5)	
Very High	668 (17.9)	223 (18.4)	445 (17.7)	
Unknown#	11	2	9	

*Values are no. (%) except as indicated. IQR, interquartile range; LGBQ, lesbian, gay, bisexual, and queer; NA, not applicable; ND, not done. †Gender categories are provided as defined by the New York City Department of Health and Mental Hygiene. ‡Because of a substantial number of persons who had unknown sexual orientation or race/ethnicity, those persons were included as a separate category in the χ^2^ test. For other characteristics, people with unknown values were excluded from χ^2^ test. §All persons who identified as Hispanic or Latino (Hispanic), regardless of race, were classified as Hispanic; all other race/ethnicity categories were non-Hispanic. ¶Neighborhood poverty level was defined as the percentage of residents in a postal (ZIP) code tabulation area with household incomes of <100% of the federal poverty level according to the American Community Survey 2016–2020 (https://www.census.gov). Neighborhoods were categorized into 4 groups: low poverty, <10% of population; medium, 10%–19.9%; high, 20%–29.9%; and very high, >30%. #Unknown because of missing residential or invalid New York ZIP code.

Mpox was diagnosed for 3,740 persons during the study period. Most mpox-positive persons were 25–44 years of age (74.7%); men (94.2%); lesbian, gay, bisexual, or queer (64.4%); Hispanic/Latino or Black/African American (61.8%); and lived in medium- or high-poverty neighborhoods (66.6%) (Table). A total of 1,213 (32.4%) persons were treated. Compared with untreated persons, a larger percentage of treated persons were lesbian, gay, bisexual, or queer (70.7% vs. 61.4%) and White (27.1% vs. 20.5%); a smaller percentage of treated persons were of unknown race/ethnicity (8.8% vs. 11.0%) or resided in Queens (11.5% vs. 18.1%) (Table).

By October 29, 2022, the percentage of treated persons was highest among those identifying as White (38.8%), then Black/African American (31.3%), Hispanic/Latino (31.0%), Asian/Pacific Islander/other (30.1%), and unknown race/ethnicity (27.7%) (Appendix Table). Percentages of treated persons were similar (30.6%–33.9%) across neighborhood poverty levels (Appendix Table). Percentages of treated persons increased initially for all racial/ethnic groups, stabilizing by late July 2022, except for White persons, among whom percentages increased an additional month before stabilizing (Figure). In all but one 2-week interval, percentages of tecovirimat-treated White persons were higher than all other groups. The increasing trend was generally consistent across neighborhood poverty levels (Figure).

**Figure:**
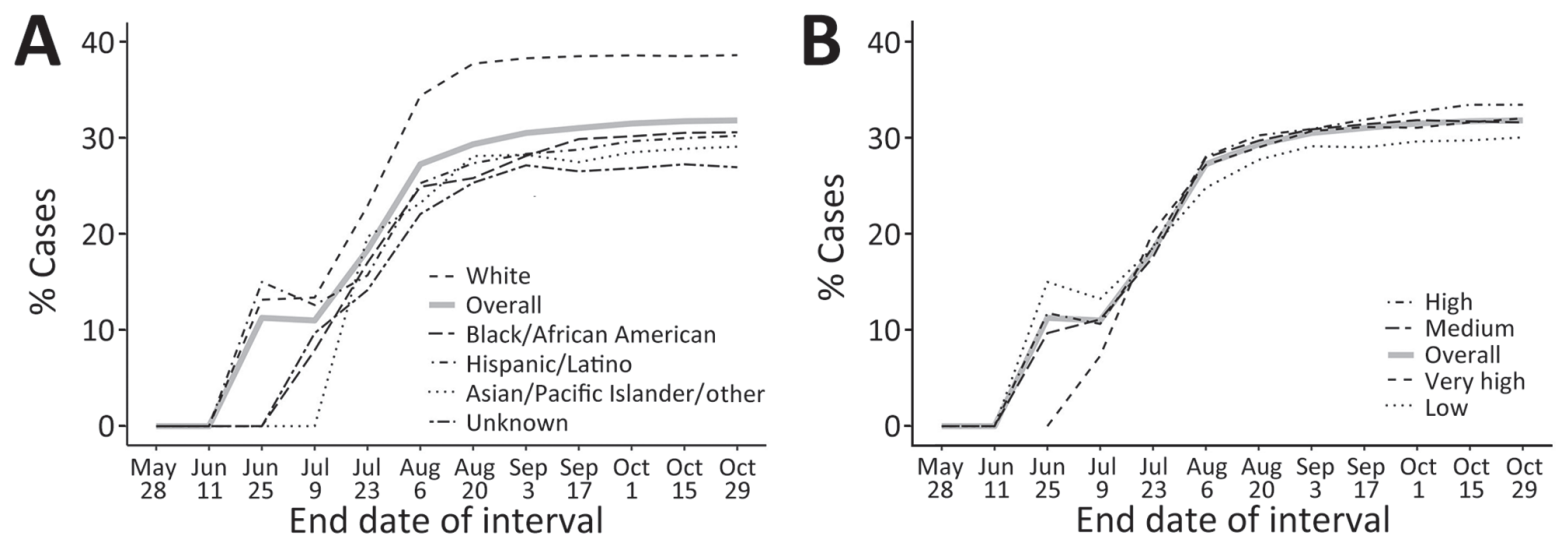
Comparisons of cumulative percentages of persons with mpox treated with tecovirimat during 2-week intervals in study of racial and socioeconomic equity of tecovirimat treatment during 2022 mpox emergency, New York City, New York, USA. Percentages of mpox cases diagnosed during May 19–October 29, 2022, are indicated. Treated persons who had no prescription date (n = 22) were not counted. A) Percentages according to race/ethnicity. B) Percentages according to neighborhood poverty level, defined as: low poverty, <10% of neighborhood population; medium, 10%–19.9%; high, 20%–29.9%; and very high, >30%.

## Conclusions

During our evaluation period, 32.4% of persons in NYC with reported mpox were treated with tecovirimat, compared with <20% nationally (*10*). The increasing percentages of treated persons during the outbreak was likely related to advocacy by and peer support from affected communities (*11*), increased prescriber familiarity with tecovirimat, and iterative revisions to the EA-IND protocol that reduced provider administrative requirements. The higher percentage of persons treated in NYC and our finding that treatment did not substantially vary by neighborhood poverty level might be attributable to the free, at-home delivery approach to tecovirimat distribution, which eliminated pharmacy access as a barrier. DOHMH also established a team to recruit and support providers to prescribe tecovirimat under the EA-IND protocol. Safety net health systems and federally qualified health centers were chosen for early outreach and technical assistance to improve access for underinsured and uninsured patients. In addition, the DOHMH team connected patients to available prescribers, if their initial providers were unable to meet EA-IND requirements.

Although percentages of tecovirimat-treated persons increased over time across all racial/ethnic groups, inequities existed. When we evaluated the cumulative percentages of treated persons in each racial/ethnic category, none approached that of White persons (≈31% for other groups vs. 38.8% for White persons) (Figure). Racial inequities and, specifically, lower percentages of treated Black/African American and Hispanic/Latino persons were foreseeable, because similar patterns have been observed for other medical countermeasures (e.g., mpox vaccines, COVID-19 antivirals, and HIV treatment) (*5*,*12*,*13*). Stigma from healthcare providers experienced by Black gay and bisexual men is a known barrier to sex-related healthcare access (*14*). Furthermore, the regulatory obligations of the EA-IND process limited the number of tecovirimat prescribers, which might have disproportionately affected Black and Hispanic/Latino communities. For example, DOHMH sexual health clinics, safety-net providers of services for Black and Hispanic/Latino men who have sex with men, did not prescribe tecovirimat until mid-September 2022 because of regulatory issues. In addition, insurance coverage inequities are a major barrier to accessing primary care (*15*), including mpox testing and treatment. No comprehensive data source identifies healthcare providers serving specific race/ethnicity groups, making interventions to increase equitable access to mpox countermeasures imprecise.

The first limitation of our study is that univariate analysis cannot capture all factors affecting treatment, such as differences in eligibility, healthcare access, and provider prescribing. Second, tecovirimat data were not available if the prescriber did not complete the online form when the drug was prescribed through a clinical trial beginning in mid-September 2022 or was dispensed from an inpatient pharmacy (e.g., some hospitalized patients). Lack of tecovirimat treatment data might have caused treatment undercounting, but we expect minimal effect because crossover between the trial recruitment period and our evaluation was brief, and the reporting form was mandatory for all prescribers.

In conclusion, our findings indicate racial inequity in tecovirimat treatment in NYC during the 2022 mpox emergency. Future responses to public health emergencies must prioritize institutional and structural racism mitigation from the outset to build more resilient communities and healthcare delivery systems. Additional analyses of factors (e.g., clinical characteristics, acceptability of treatment, detailed sociodemographic information) should be prioritized to assess the extent and effect of race/ethnicity on mpox treatment distribution and to inform future efforts to achieve equitable medical countermeasure access. Having comprehensive data for race/ethnicity of populations served by healthcare providers/networks and for characteristics of persons receiving medical countermeasures is critical for improving equity in emergency preparedness and response. Although neither dataset is sufficient to overcome institutional or structural racism, the alternative, a reactive approach, will inevitably perpetuate entrenched inequities.

Appendix Additional information for racial and socioeconomic equity of tecovirimat treatment during the 2022 mpox emergency, New York, New York, USA.
