# Supplementary material for: Racial and Socioeconomic Equity of Tecovirimat Treatment during the 2022 Mpox Emergency, New York, New York, USA
Source: Emerg Infect Dis. 2023 Nov;29(11):2353–7. doi: 10.3201/eid2911.230814 (PMC10617352; doi:10.3201/eid2911.230814)
Supplement: Appendix — Additional information for racial and socioeconomic equity of tecovirimat treatment during the 2022 mpox emergency, New York, New York, USA. [file 23-0814-Techapp-s1.pdf]

Article DOI: https://doi.org/10.3201/eid2911.230814

*EID cannot ensure accessibility for supplementary materials supplied by authors. Readers who have difficulty accessing supplementary content should contact the authors for assistance.*

# Racial and Socioeconomic Equity of Tecovirimat Treatment during the 2022 Mpox Emergency, New York, New York, USA

## Appendix

**Appendix Table.** Cumulative percentages of persons with mpox treated with tecovirimat during 2-week intervals in study of racial and socioeconomic equity of tecovirimat treatment during 2022 mpox emergency, New York City, New York, USA, May 19, 2022–October 29, 2022*

| | | Race/ethnicity | | | | | Neighborhood poverty level† | | | |
|---|---|---|---|---|---|---|---|---|---|---|
| End dates | Overall | Asian/Pacific Islander/other | Black/African American | Hispanic/ Latino | White | Unknown | Low | Medium | High | Very high |
| May 28 | 0 | 0 | 0 | NI | 0 | 0 | 0 | 0 | 0 | NI |
| Jun 11 | 0 | 0 | 0 | 0 | 0 | 0 | 0 | 0 | 0 | NI |
| Jun 25 | 11.3 | 0 | 0 | 15.0 | 13.2 | 0 | 15.0 | 9.7 | 11.8 | 0.0 |
| Jul 9 | 11.0 | 0 | 7.9 | 12.6 | 13.4 | 9.7 | 13.3 | 11.1 | 10.6 | 7.3 |
| Jul 23 | 18.3 | 19.7 | 17.0 | 15.7 | 22.9 | 14.2 | 18.6 | 17.6 | 18.7 | 20.2 |
| Aug 6 | 27.3 | 23.2 | 24.9 | 25.3 | 34.4 | 22.1 | 24.8 | 28.0 | 28.0 | 27.2 |
| Aug 20 | 29.3 | 28.1 | 25.8 | 27.4 | 37.7 | 25.3 | 27.7 | 29.7 | 30.3 | 29.0 |
| Sep 3 | 30.5 | 28.2 | 28.1 | 28.3 | 38.3 | 27.1 | 29.1 | 30.9 | 30.9 | 30.7 |
| Sep 17 | 31.0 | 27.5 | 29.9 | 28.8 | 38.5 | 26.5 | 29.0 | 31.4 | 31.9 | 31.1 |
| Oct 1 | 31.5 | 28.5 | 30.2 | 29.7 | 38.6 | 26.8 | 29.6 | 31.8 | 32.7 | 31.1 |
| Oct 15 | 31.7 | 28.9 | 30.5 | 30.0 | 38.5 | 27.2 | 29.7 | 31.7 | 33.4 | 31.6 |
| Oct 29 | 31.8 | 29.1 | 30.6 | 30.2 | 38.6 | 26.9 | 30.1 | 31.6 | 33.4 | 32.0 |
| Total‡ | 32.4 | 30.1 | 31.3 | 31.0 | 38.8 | 27.7 | 30.6 | 32.0 | 33.9 | 33.4 |

*End dates of 2-week intervals are indicated. NI, no identified persons with mpox.
†Poverty levels were defined as the percentage of residents in the patient's ZIP code living below the federal poverty level according to the American Community Survey (https://www.census.gov). Neighborhoods were categorized by using the NYC DOHMH EpiQuery tool (https://a816-health.nyc.gov/hdi/epiquery) into 4 groups: low poverty, <10% of population; medium, 10%–19.9%; high, 20%–29.9%; and very high, ≥30%.
‡Total percentages include treated persons who had no prescription date (n = 22) and, therefore, could not be assigned to a 2-week interval period.
